# Aligning Perspectives: Autism Identity, Independence, Participation, and Quality of Life in Autistic Adolescents Through Self and Parental Reports

**DOI:** 10.1007/s10803-025-06836-6

**Published:** 2025-04-19

**Authors:** Liron Lamash, Yael Gutman, Sonya Meyer, Eynat Gal

**Affiliations:** 1 Department of Occupational Therapy, Faculty of Social Welfare & Health Sciences, University of Haifa, Haifa, Israel; 2 Department of Occupational Therapy, Faculty of Health Sciences, Ariel University, Ariel, Israel

**Keywords:** Autism spectrum disorder, Adolescents, Autism identity, Functional autonomy, Social participation, Quality of life

## Abstract

This study examines the alignment between self-reports and parental reports on adolescents’ autism identity, functional autonomy, social participation, and quality of life (QoL), providing insights into the relationships between these factors. Thirty dyads comprising adolescents aged 13 to 18 years and one of their parents participated in the study. Participants completed the Autism Identity Questionnaire, Daily Routine and Autonomy questionnaire, Child and Adolescent Scale of Participation-Youth, and the Pediatric Quality of Life Inventory. The findings indicated moderate agreement between self-reports and parental reports regarding autism identity, functional autonomy, and QoL. Fair agreement was found regarding social participation. Adolescents who reported higher levels of acceptance regarding their autism identity demonstrated greater autonomy and participation, which correlated with a higher QoL. Conversely, those with higher engulfment scores (feeling overwhelmed by the diagnosis) exhibited lower levels of independence, social participation, and emotional and social QoL. These findings suggest that fostering a positive autism identity may enhance autonomy and social participation while addressing feelings of engulfment could improve emotional and social outcomes.

## Introduction

Autistic individuals experience differences in social communication, interaction, and sensory processing, which shape their daily functioning across social, emotional, and occupational domains (American Psychiatric & Association, 2013). While research on autism has expanded in recent years, studies explicitly focusing on autistic adolescents and young adults—particularly their perspectives—remain limited (Goldman & Preece, 2023; Mercado-Garrido et al., 2024; Volkmar et al., 2024).

Adolescence represents a pivotal developmental stage characterized by significant growth and transformation. This period involves the formation of identity, the adoption of social roles, and the development of skills necessary for mature interpersonal relationships. It also includes the emergence of abstract thinking and the progression toward social and economic autonomy (Curtis, 2015; Sawyer et al., 2018; World Health Organization, 2021). Adolescents gradually separate from their parents, assert their independence, and work towards establishing a distinct self-identity (Erikson, 1994). For autistic adolescents, however, these developmental milestones can be particularly challenging due to inherent social and communicative difficulties, which may exacerbate the struggle for independence and complicate identity formation (Husmann et al., 2024; Kapp et al., 2011; Lombardo et al., 2009). Some autistic individuals may perceive their diagnosis negatively and seek to distance themselves from the autism label, while others may embrace their diagnosis and focus on their strengths (K. Cooper et al., 2017). Understanding these diverse perspectives is essential for providing effective support to autistic adolescents as they navigate the complex interplay between identity, independence, participation, and overall quality of life (QoL).

Identity is a multifaceted concept encompassing various aspects of an individual’s self, including traits, personal characteristics, roles, physical appearance, values, goals, and past experiences (Dunn & Burcaw, 2013). While identity develops as a lifelong process, it becomes particularly significant during adolescence and young adulthood, a critical period for self-discovery and personal growth (Arnett, 2000; Erikson, 1994; Sugimura et al., 2023). A well-defined self-identity enables individuals to engage in goal-directed and autonomous behavior. It enables individuals to recognize their strengths and limitations, fostering a belief in their ability to control their lives and function successfully in society (Crocetti et al., 2023; Field et al., 1998). Those with a well-defined self-identity are more likely to engage in activities promoting their personal growth and achievement, enhancing their psychological well-being (Campbell et al., 2003; Carter et al., 2010; Garberoglio et al., 2017).

Self-identity is significantly shaped through social interactions within society (Christiansen, 1999; Pérez-Torres, 2024). Therefore, the development of self-identity presents unique challenges for autistic adolescents and young adults. Despite its importance for coping with diagnosis, readiness for treatment, and daily functioning, aspects of self-identity in autistic adolescents have not been adequately studied (Holmbeck, 2002; Oris et al., 2016; Williams et al., 2002). Understanding these identity-related challenges can empower autistic individuals to grasp their limitations better and independently explore their identities (Dunn & Burcaw, 2013).

The term *illness identity* has emerged in recent years to describe how a health or developmental condition integrates with an individual’s overall identity and daily life (Oris et al., 2016). This concept is useful for understanding why some individuals with chronic illnesses struggle to cope while others adapt more successfully. Researchers like Oris et al. (2016) have identified four dimensions of illness identity: (a) *rejection*—the rejection of the disease by the individual and its non-inclusion in their identity; (b) *engulfment*—the overwhelming feeling that the disease impacts one’s identity and controls their life; (c) *acceptance*—the extent to which the person accepts their illness as a part of their identity; and (d) *enrichment*—the degree to which the person believes that the disease has allowed them to develop and sees advantages in it within the context of their identity. Rejection and engulfment are considered negative dimensions of identity perception, while acceptance and enrichment are viewed as positive (Van Bulck et al., 2019). Given that autism is a lifelong neurodevelopmental condition rather than an illness, the term “illness identity” has been adapted to “autism identity” to more accurately reflect how autism is integrated into an individual’s sense of self.

*Quality of life* encompasses various dimensions of well-being, including physical, emotional, and social aspects. It is often influenced by individuals’ ability to engage in meaningful daily activities and social interactions (Varni et al., 2001). Although QoL in autistic adolescents has been extensively studied relative to daily functioning (e.g. Kamp-Becker et al., 2010; McCauley et al., 2020; Ozboke et al., 2022) and social participation (e.g. DaWalt et al., 2019; Hilton et al., 2008; Potvin et al., 2015), the relationship between autistic adolescents’ QoL and their perception of their diagnosis has received limited attention (Lamash & Meyer, 2022; Lamash et al., 2024). Understanding this relationship could offer valuable insights for developing targeted interventions to improve QoL for autistic adolescents.

Research examining the disparities between parental and child reports has shown that parents tend to agree more with their children on objective external measures, such as physical activities like walking, running, school attendance, and aggressive behaviors. However, there is less agreement on internal measures, such as emotions, worry, emotional stress, and physical sensations like fatigue, pain, and difficulties (Jozefiak et al., 2008; Patel et al., 2017; Sheldrick et al., 2012).

These differences can be attributed to the limitations of parental reporting, often based on what the child communicated to them or what they observe. Conversely, self-reporting captures the child’s subjective experiences. Additionally, the parents’ mental state and personal experiences can influence their reports, which may not accurately reflect their child’s experiences (Eiser & Varni, 2013; Lagattuta et al., 2012). In some instances, mothers’ reports of their children’s QoL align with the children’s assessments, highlighting that the degree of agreement can vary depending on the context (Eiser & Varni, 2013).

Similar discrepancies are observed between self-reports and parental reports for autistic children and adolescents across various domains. These differences may reflect variations in focus, with the parents emphasizing observable behaviors whereas the adolescents highlight their internal experiences, such as emotions and anxiety (Clark et al., 2015; Patel et al., 2017; Sheldrick et al., 2012). Additionally, some adolescents engage in camouflaging behaviors to align with social expectations, which can further impact self-reports (Hull et al., 2019; Keith et al., 2019). On the other hand, parental reports may be biased by the mental health challenges commonly experienced by parents of autistic children, such as stress, anxiety, and depression (Uljarević et al., 2017). Despite these challenges, self-reporting by autistic adolescents, particularly those with higher cognitive abilities, is increasingly recognized as valuable and important for the development of autonomy during this period (Lamash et al., 2020; Sheldrick et al., 2012). The distinct perspectives offered by self-reports and parental reports underscore the importance of gathering data from both sources because each provides unique insights and limitations (Clark et al., 2015; Keith et al., 2019). Therefore, this study aimed to explore how autistic adolescents and their parents perceive key aspects of the adolescents’ lives, including autism identity, functional autonomy, social participation, and QoL. Specifically, this study aimed to:

1. Evaluate the agreement between adolescents’ self-reports and their parents’ reports on autism identity, functional autonomy, social participation, and QoL.
2. Examine the relationship between the adolescents’ perceptions of their autism identity, functional autonomy, participation, and QoL.

## Methods

### Study Design

In this quantitative, cross-sectional study, data were collected at a single point in time, providing a snapshot of the relationships between the variables. Participants were recruited using a convenience sampling method.

### Participants

An a priori power analysis (conducted using G*Power) indicated that 64 participants (32 per group) were needed to achieve adequate power (0.8) with an alpha level of 0.05. The final sample included 30 dyads of autistic adolescents and one of their parents. The adolescents were aged 13 to 18 years (*M* = 16.32, *SD* = 1.42), 27 of whom identified as male (90%) and three identified as female (10%). Based on information obtained from school records or parent reports, all participants had typical intellectual functioning (IQ within the average range), had been classified as requiring Level 1 support for verbal communication (indicating minimal support needs), and had sufficient reading and writing skills to independently complete the self-report questionnaires and provide informed consent for participation in the study. However, no information was collected regarding support levels in other social communication or adaptive functioning aspects. All adolescents had been diagnosed between the ages of 1 and 14 years (*M* = 7.70, *SD* = 4.42) and were informed of their diagnosis between the ages of 6 and 17 years (*M* = 11.88, *SD* = 2.73). The time gap between when parents received the official diagnosis and when they disclosed the autism diagnosis to their child ranged from 0 to 12 years (*M* = 4.20, *SD* = 3.79). Educational placements included 16.7% in regular classrooms, 6.7% in regular classrooms with an aide, 66.7% in specialized communication classrooms within mainstream schools, and 10% in special education frameworks. Most (90%) participants lived in urban areas, and 10% lived in rural areas. The parent group comprised 25 mothers (83%) and five fathers. Regarding parental education, 63.3% of mothers had an academic degree, 16.7% had professional training, and 20% had completed high school. Similarly, 50% of fathers had an academic degree, 30% had professional training, and 20% had completed high school.

### Measures

Both self-report versions for adolescents and parent-report versions were used for all questionnaires used in the methodology.

#### Demographic Questionnaire

Parents provided basic demographic information about their child, including age, gender, and age at diagnosis. Additionally, parents were asked to provide their gender and age.

#### Illness Identity Questionnaire

The Illness Identity Questionnaire (IIQ; Oris et al., 2016) assesses how much an individual’s health or developmental condition integrates with their identity and daily life. With the IIQ author’s authorization, the IIQ was adapted for autistic individuals. The IIQ consists of 25 items rated on a 5-point scale from 1 (*completely disagree*) to 5 (*completely agree*). It measures four dimensions of diagnostic identity: (a) rejection, (b) engulfment, (c) acceptance, and (d) enrichment. A mean and a total score are calculated for these four dimensions. A higher mean score reflects a more positive perception of illness identity. The IIQ has demonstrated good internal consistency and validity among adolescents with various chronic illnesses (Oris et al., 2016) and has shown strong construct validity and good internal consistency among autistic adolescents and young adults (*N* > 100; ω = 0.85–0.90; Lamash & Meyer, 2025). In the current study, parents completed a similar form and were asked to assess how their child perceived having autism. The internal consistency in the current sample was α = 0.76 for the self-report version and α = 0.74 for the parent-report version. The slightly lower internal consistency in the current sample compared to the previous larger dataset is likely due to the smaller sample size and restricted response variance.

#### Daily Routine and Autonomy Questionnaire

The Daily Routine and Autonomy (DRA; Lamash & Josman, 2021) questionnaire assesses individuals’ level of autonomy in performing daily activities and their desire for independence. The DRA consists of 31 items across three domains: (a) basic self-care and routine activities, (b) complex or multistage daily activities, and (c) social and leisure activities. For each item, respondents rate their level of and desire for independence on a scale from 1 (*completely independent; very important for me to be independent*) to 3 (*not independent at all; not important to me to be independent*). The DRA has demonstrated good construct validity and high internal consistency among autistic adolescents, with Cronbach’s alpha coefficients typically exceeding 0.80 (Lamash & Josman, 2021). In the DRA parent-report version, parents assess their child’s level of independence from their perspective and their desire for the child’s increased independence in various activities. The internal consistency in the current sample was α = 0.84 for the self-report version and α = 0.92 for the parent-report version.

#### Child and Adolescent Scale of Participation-Youth

The Child and Adolescent Scale of Participation-Youth (CASP-Y; Bedell, 2009) self- and parent-report measures the extent to which adolescents participate in home, school, and community activities compared to their peers. The items and rating scales are identical in both versions. The CASP-Y includes 20 items divided into four sections: (a) home participation, (b) neighborhood and community participation, (c) school participation, and (d) home and community living activities. Each item is rated on a scale from 1 to 4 (4 = *age-expected/full participation*, 3 = *somewhat restricted*, 2 = *very restricted*, and 1 = *unable*), with an additional option for *not applicable*. Mean scores are calculated for total and subscale scores, with higher scores indicating greater age-expected participation. The CASP-Y demonstrates strong internal consistency (α = 0.90), test–retest reliability (α = 0.94), and construct validity (Bedell, 2004, 2009). It also has an internal consistency of 0.87 among adolescents with various disabilities, including autism (McDougall et al., 2013). The internal consistency in the current sample was α = 0.95 for the self-report version and α = 0.91 for the parent-report version.

#### Pediatric Quality of Life Inventory

The Pediatric Quality of Life Inventory (PedsQL; Varni et al., 2001) was developed to assess the health-related QoL in children and adolescents aged 2 to 18 years with and without various health conditions. The PedsQL consists of 23 items measuring four functional metrics: physical, emotional, social, and school. These items are rated on a 5-point scale, ranging from 0 (*never a problem*) to 4 (*always a problem*). The scores are then converted to a linear score on a scale of 0 to 100, where higher scores indicate a better QoL. The PedsQL has demonstrated good internal reliability for self- and parental reporting (α = 0.88 and α = 0.90, respectively) and high internal consistency in autistic adolescents (α = 0.93; Shipman et al., 2011). Additionally, the PedsQL has good structural validity and discriminates between groups with different levels of morbidity, such as those with chronic disease, acute illness, and healthy individuals (Varni et al., 2001). The PedsQL can be completed through self- and parent-report versions, allowing for a comprehensive assessment of the child’s quality of life from both perspectives. The Hebrew version of the PedsQL questionnaire was used in this study, and access was obtained through the Mapi Research Trust. The internal consistency in the current sample was α = 0.91 for the self-report version and α = 0.91 for the parent-report version.

### Procedure

The study received approval from the University of Haifa’s Ethics Committee and the Chief Scientist at Ministry of Education in Israel. We conducted recruitment through educational settings for autistic adolescents, including inclusive regular classrooms, specialized classrooms for autistic students within regular schools, and special education schools. School staff distributed an online information letter to parents of potential participants detailing the study and its importance. Adolescents and their parents who were interested in participating provided online informed consent and completed the questionnaires on the institutional Google Forms platform, which is protected by two-factor authentication to ensure data security and confidentiality. Responses were submitted anonymously and automatically transmitted to the database. The dyads were matched using a serial number and the adolescent’s initials. Google Forms saved only fully completed questionnaires, preventing our assessment of the number of participants who dropped out during the questionnaire process.

### Data Analysis

The data were analyzed using IBM SPSS (Version 27.0). Cronbach’s alpha (α) was used to assess the tools’ internal consistency in the current sample. Descriptive statistics were calculated for the study population and measurements. An intraclass correlation coefficient (ICC) analysis was performed to evaluate the agreement between adolescents’ and parents’ ratings. A two-way mixed-effects model with an absolute agreement definition was used to determine the ICC values. A paired *t*-test and Cohen’s *d* assessed differences between adolescent and parent responses on the IIQ. Pearson correlation analyses examined associations between autism identity perception, functional autonomy, social participation, and quality of life.

## Results

### Agreement Between Self- and Parental Reports

#### Autism Identity

The ICC for single measures was found to be 0.60 (95% CI [0.31, 0.79]), indicating moderate agreement between individual parent and child scores. This suggests that while there is a noticeable degree of concordance between the two groups, variability remains between individual responses. For average scores, the ICC was higher, with a value of 0.75 (95% CI [0.48, 0.88]), reflecting a good level of agreement when averaging the scores across all dimensions. This suggests that parent and child responses are more closely aligned when considering the overall scores. The significance test associated with both ICC values was statistically significant (*p* < 0.001), confirming the level of agreement.

**Fig. 1:**
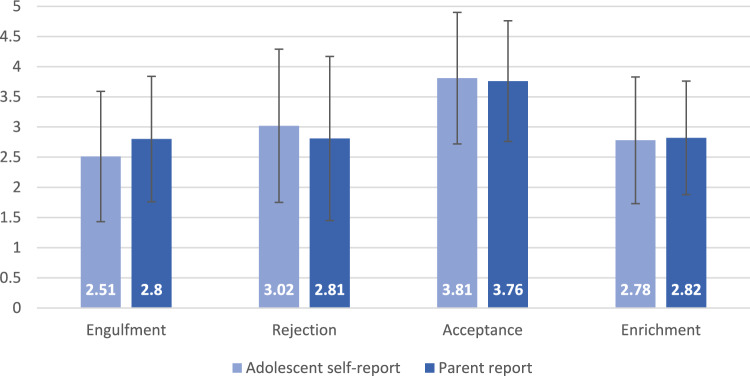
Adolescents’ and Parents’ self-reports of autism identity perception as expressed in the Illness Identity Questionnaire (IIQ)

A paired-sample* t*-test indicated no significant difference between self- and parental reports in the overall IIQ score *t*(29) = − 0.47, *p* = NS, *d* = 0.06. No significant differences were found between adolescent self-reports and their parent reports in the IIQ categories of rejection, acceptance, engulfment, or enrichment. These results suggest that the parents described their child’s autism identity perception with minimal discrepancies with the adolescents’ self-report. Figure 1 presents the means and standard deviations of the IIQ for self- and parental reports on the adolescents’ autism identity perceptions.

#### Functional Autonomy

An ICC analysis for DRA independence level scores for single measures was 0.44 (95% CI [0.01, 0.71]), indicating moderate agreement between the adolescents and parent scores. The wide confidence interval suggests variability in the degree of agreement across the sample. The ICC was higher for average scores, with a value of 0.61 (95% CI [0.02, 0.83]), reflecting a good agreement between the adolescents’ and parents’ average scores. Both ICC values were statistically significant (*p* < 0.001). However, the ICC for single measures of desire for independence was 0.04 (95% CI [− 0.26, 0.36]), indicating a very low level of agreement between individual parent and child scores. The ICC was slightly higher for average scores but still very low, at 0.09 (95% CI [− 0.68, 0.53]). Neither the single measures nor the average score ICC values were statistically significant (*p* = 0.39).

A paired-samples *t*-test indicated significant differences between the adolescents’ and parents’ reports for *independent level*, *t*(29) =  − 4.53, *p* < 0.001, *d* = 0.83, as well as for *desire for independence*, *t*(28) = 2.38, *p* < 0.05, *d* = 0.60, indicating that adolescents reported higher independence levels and less desire for independence than their parents reported. Table 1 presents the DRA categories’ descriptive statistics and paired-sample *t*-test results.

**Table 1:** Daily routine and autonomy (DRA) questionnaire descriptive statistics and paired-sample T-test results

DRA category	Adolescent self-report		Parent report		*t*	*p*	Cohen’s *d*
	Range	*M* (*SD*)	Range	*M* (*SD*)			
Level of independence							
Basic self-care and routine activity	1.00–1.82	1.29 (.26)	1.09–2.45	1.54 (.35)	− 3.96	< .001	.81
Complex or multistage daily activity	1.00–2.70	1.50 (.45)	1.10–2.90	1.76 (.46)	− 5.33	< .001	.57
Social and leisure activity	1.00–1.86	1.29 (.31)	1.00–2.14	1.52 (.35)	− 3.86	< .001	.70
Total independence	1.00–2.06	1.33 (.27)	1.16–2.42	1.55 (.31)	− 4.52	< .001	.77
Desire for independence							
Basic self-care and routine activity	1.00–2.27	1.34 (.39)	1.00–1.64	1.10 (.14)	3.11	.004	.83
Complex or multistage daily activity	1.00–2.80	1.43 (.51)	1.00–2.20	1.25 (.32)	1.72	.096	.42
Social and leisure activity	1.00–2.14	1.27 (.34)	1.00–1.86	1.13 (.24)	1.81	.080	.47
Total desire for independence	1.00–2.29	1.33 (.37)	1.00–1.71	1.15 (.19)	2.38	.024	.60

A DRA low mean score represents a higher level of independence and more desire for independence

#### Social Participation

An ICC analysis for CASP-Y scores for single measures yielded a value of 0.39 (95% CI [0.06, 0.65]), indicating fair agreement between adolescents and parent reports. The wide confidence interval reflects variability in agreement across the sample. The ICC was higher for average measures, with a value of 0.56 (95% CI [0.11, 0.79]), suggesting moderate agreement. Both ICC values were statistically significant (*p* < 0.01), indicating that despite variability, there is a meaningful correlation between the self-reports and parent reports in this context.

A paired *t*-test revealed a significant difference between adolescent self-reports and parent reports regarding total social participation, *t*(28) = 2.88, *p* < 0.01, *d* = 0.54, with adolescents reporting significantly higher participation levels than their parents reported. Table 2 presents the CASP-Y categories’ descriptive statistics and paired-sample* t*-test results.

**Table 2:** The Child and Adolescent Scale of Participation-Youth (CASP-Y) categories’ descriptive statistics and paired-sample T-test results

CASP-Y Category	Adolescent self-report		Parent report		*t*	*p*	Cohen’s *d*
	Range	*M* (*SD*)	Range	*M* (*SD*)			
Home	17–24	22.03 (2.15)	13–24	20.14 (2.98)	3.96	< .001	.73
Neighborhood and community	4–16	11.76 (3.80)	4–16	10.79 (2.94)	1.24	.23	.28
School	12–20	18.24 (2.12)	10–20	17.76 (2.42)	0.91	.37	.21
Home and community living activity	7–20	16.66 (3.49)	7–20	15.45 (3.62)	2.59	.02	.34
Total participation	41–80	68.69 (9.39)	40–80	64.14 (9.53)	2.88	.01	.48

#### Quality of Life

An ICC analysis for PedsQL scores using a two-way mixed-effects model showed an ICC of 0.49 (95% CI [0.17, 0.72], *p* = 0.001) for single measures, indicating moderate agreement between the two sources of reports. The ICC for average measures was higher, at 0.66 (95% CI [0.30, 0.84], *p* = 0.001), reflecting a moderate yet stronger agreement when averaging the reports.

A paired *t*-test revealed a significant difference between adolescent self-reports and parent reports, *t*(28) = 2.24, *p* = 0.03, *d* = 0.41, with adolescents reporting higher overall QoL scores than their parents. Table 3 presents the QoL categories’ descriptive statistics and paired-sample *t*-test results.

**Table 3:** Pediatric quality of life inventory (PedsQL) categories’ descriptive statistics and paired-sample *T*-test results

PedsQL category	Adolescent self-report		Parent report		*t*	*p*	Cohen’s *d*
	Range	*M* (*SD*)	Range	*M* (*SD*)			
Physical health	3.1–96.9	65.83 (20.86)	6.2–96.9	56.56 (25.33)	2.11	.04	.39
Emotional functioning	5.0–95.0	54.50 (21.43)	20.0–100.0	52.50 (21.28)	0.52	.61	.09
Social functioning	10.0–100.0	69.17 (21.94)	10.0–100.0	54.17 (22.05)	4.01	< .001	.73
School functioning	15.0–95.0	59.50 (18.77)	10.0–100.0	59.00 (20.78)	0.13	.90	.02
Total	9.8–92.4	62.72 (17.11)	25.0–97.8	55.69 (18.03)	2.24	.03	.41

### The Relationship Between Autism Identity Perception and Functional Autonomy, Participation, and Quality of Life

Only the adolescents’ self-reported data were used to assess the associations between their perceptions of autism identity and various health-related outcomes, including functional autonomy, social participation, and QoL.

#### Functional Autonomy

No significant correlations were found between the adolescents’ overall perception of their autism identity, their total independence in daily activities, or their desire for independence. However, analyses of correlations between the autism identity dimensions and the functional autonomy categories revealed that the engulfment dimension significantly correlated to the desire for independence in basic self-care and routine activities (*r* = − 0.36, *p* < 0.05). Specifically, adolescents who reported higher levels of engulfment by their autism identity expressed less desire for independence in self-care and routine activities. In contrast, the acceptance dimension significantly correlated to the desire for independence in social and leisure activities (*r* = 0.37, *p* < 0.05), indicating that adolescents who reported greater acceptance of their autism identity expressed a stronger desire for independence.

#### Social Participation

No significant correlation was found between the adolescents’ perception of their autism identity and their reported levels of social participation. Furthermore, no significant correlations were found between the various dimensions of autism identity and the different social participation environments.

#### Quality of Life

A significant positive correlation was found between the adolescents’ autism identity perception and their QoL (*r* = 0.38, *p* < 0.05). The more positive their autism identity perception was, the better QoL they reported. Further analyses of correlations between the autism identity dimensions and the PedsQL categories revealed that the engulfment dimension negatively correlated to their emotional (*r* = − 0.43, *p* < 0.05) and social (*r* = − 0.40, *p* < 0.05) QoL. Specifically, adolescents who reported higher levels of engulfment by their autism identity expressed lower emotional and social QoL.

## Discussion

The current study provides valuable insights into the autism identity perception of autistic adolescents and its relationships with their functional autonomy, social participation, and overall QoL. Understanding the implications of autism identity formation and the discrepancies between adolescent and parental perceptions underscores the importance of these factors in the broader context of evaluation and intervention processes for autistic adolescents.

The findings indicate a relatively balanced distribution across the IIQ dimensions of how autistic adolescents perceive their condition as part of their overall identity. There is a notable trend towards acceptance of autism as a part of their identity, as reflected by higher scores in the acceptance dimension than engulfment or rejection. This suggests that the current study participants successfully integrated their diagnosis into a positive sense of self, aligning with existing literature demonstrating the benefits of a positive autism identity for social and emotional well-being (K. Cooper et al., 2017; Oris et al., 2016). Cage et al. (2018) further found that many autistic adolescents and adults view their autism positively, identifying it as a source of personal strengths, such as focus, determination, and unique ways of thinking. These positive self-perceptions are crucial for fostering resilience and mitigate the negative impact of societal stigma on autistic individuals. This aligns with the current study’s results, demonstrating that acceptance is the strongest component of autism identity, reflecting the growing recognition of neurodiversity as a source of strength. However, these findings may not be representative of all autistic adolescents, particularly those who face greater challenges in accepting their diagnosis. Furthermore, although these results suggest a potential relationship between autism identity and well-being, they should be interpreted with caution. Further research is needed to explore additional factors that may influence these associations.

The engulfment dimension, which measures the extent to which an individual feels overwhelmed by the diagnosis, remained significantly present among autistic adolescents and cannot be ignored. Although they may perceive their autism positively, adolescents still experience struggle with the dominance of autism in their identity, potentially leading to diminished emotional and social QoL. These findings underscore the need for tailored interventions that assist adolescents in navigating the complexities of their autism identity by reducing feelings of engulfment and promoting the positive aspects of their autism (Cage et al., 2018).

The moderate agreement between adolescent self-reports and parental reports on the IIQ highlights the importance of integrating both perspectives into assessment and intervention planning. It is essential for parents not only to report their child’s functional capabilities but also to gain a clear understanding of how their child perceives their autism. Parental insight into the adolescent’s identity formation is essential for fostering a supportive environment that promotes positive identity development. When parents understand their child’s self-perception, they can provide more targeted and empathetic support, enhancing the adolescent’s emotional and social well-being (Kirby & Hodges, 2018).

While the benefits of self-reporting are evident, particularly in offering a direct glimpse into the adolescent’s subjective experience, there are instances where parental reports prove invaluable. This is particularly true when adolescents encounter challenges in articulating or reflecting on their experiences, a common difficulty among some autistic individuals, especially those with limited communicative abilities (Shipman et al., 2011). In such cases, parental input can bridge the gaps, offering insights that the adolescent may be unable to express. Moreover, parents can provide an external perspective on behaviors and functional abilities that might be overlooked in self-assessment, ensuring a more comprehensive evaluation of the adolescent’s needs (Stokes et al., 2017).

### Functional Autonomy

The descriptive findings from the DRA questionnaire reveal a complex relationship between perceived levels of independence and the desire for greater autonomy among autistic adolescents. The adolescents reported relatively high levels of independence in basic self-care and routine activities. However, they did not express a desire for greater autonomy in these areas, suggesting they may be content with their current functioning level, experience apprehension, or lack confidence with more complex aspects of daily life (Hume et al., 2021).

The DRA also highlights disparities in more complex or multistage activities, such as social or leisure activities. Here, adolescents reported lower levels of independence but demonstrated a moderate desire to increase autonomy. This indicates that although adolescents may recognize their limitations in these more advanced functional domains, they are also willing to develop greater independence. Such findings align with broader research suggesting that autistic adolescents often struggle more with socially driven activities yet exhibit a desire for improvement in these areas, particularly as they approach adulthood and seek greater self-determination (Gotham et al., 2015).

Additionally, the DRA revealed marked differences between adolescents’ self-reports and parental reports. Adolescents consistently rated themselves as more independent than their parents perceived them, particularly in complex or multistage daily activities and social and leisure activities. This gap between self-perception and parental perception may arise from several factors. Parents often adopt a protective stance, sometimes underestimating their child’s capabilities due to concerns about safety, social norms, or the adolescent’s ability to manage independently in challenging situations (Chen et al., 2018). Alternatively, adolescents may overestimate their independence due to a desire for social conformity or a limited ability to assess their functioning accurately. Research has shown that self-assessments by autistic adolescents can sometimes reflect an overestimation of abilities, especially in areas requiring social or cognitive judgment (Cheak-Zamora et al., 2015). These differences in perception highlight the importance of integrating self- and parental reports when assessing functional independence to ensure a comprehensive and balanced understanding of the adolescent’s true capabilities and the areas where additional support may be needed.

### Social Participation

The findings derived from the CASP-Y assessment indicate that adolescents exhibit moderate levels of social participation across various environments; however, these levels are notably lower than those of their neurotypical peers. These findings reflect the challenges many autistic adolescents encounter in social settings, particularly during unstructured social interactions or situations that demand more social complexity (Hilton et al., 2008; Potvin et al., 2013).

A key finding from our study is the discrepancy between adolescent self-reports and parental assessments regarding social participation. Adolescents frequently rated their participation levels higher than their parents, particularly in community and social activities. This divergence may reflect differences in how each group perceives and interprets participation. Adolescents may focus on their internal social interaction experiences, whereas parents often base their reports on visible social behaviors and challenges (Eiser & Varni, 2013; Stokes et al., 2017). Moreover, driven by optimism or social desirability, adolescents may wish to present themselves as more socially active than they actually are, which can further contribute to the gap between self-reports and parental reports (Cheak-Zamora et al., 2015; Clark et al., 2015).

Conversely, parents may report lower participation levels due to their observations of specific social deficits or challenges the adolescent may not fully recognize. Possibly, discrepancies between the parent and adolescent reports arise from parents not fully recognizing autistic communication styles or interpreting their social participation based on conventional social norms (Kapp, 2020; Milton, 2012). Concerns about their child’s safety and social well-being often shape parental perceptions, leading parents to report their adolescent’s abilities to navigate social environments more conservatively than their children do (Chen et al., 2018; Lerner et al., 2017). These discrepancies underscore the importance of integrating both perspectives into assessments because relying solely on self-reports or parental reports may result in an incomplete understanding of the adolescent’s social functioning (L. Jones et al., 2015).

### Quality of Life

This study’s findings reveal positive and challenging aspects of the adolescents’ QoL. The autistic adolescents reported moderate to high overall QoL, particularly in the domains of emotional and social functioning. These self-reports suggest a generally positive outlook on life, consistent with existing literature that has indicated many autistic adolescents express satisfaction with their daily routines and social interactions despite facing associated challenges (McCauley et al., 2020). This relatively positive perception may indicate a sense of contentment within their social world, often shaped by their unique experiences and interactions, differing from the expectations or norms of neurotypical peers (Falkmer et al., 2015).

However, a consistent finding across this study is that adolescents’ self-reports were frequently more optimistic than those provided by their parents. Parents tended to rate their children’s QoL lower, particularly in social and emotional functioning domains. This discrepancy aligns with previous research, which indicates that autistic adolescents often perceive their QoL more positively than their parents, possibly due to differences in how they internalize or express social and emotional challenges (Potvin et al., 2015). Autistic adolescents may emphasize their subjective sense of well-being, which may not always align with the external difficulties they face, such as challenges in social participation or emotional regulation. However, social difficulties may not necessarily prevent them from reporting high QoL; self-reported well-being can reflect areas of satisfaction beyond conventional social expectations (Shipman et al., 2011).

In contrast, parents are more likely to be attuned to visible challenges, such as difficulties in forming peer relationships or navigating social environments, which often results in lower ratings of their child’s QoL (Davis & Carter, 2008). By integrating both viewpoints, a more comprehensive understanding can be achieved. This integration enables the development of interventions that address adolescents’ subjective experiences while acknowledging the observable challenges identified by their parents (Sheldrick et al., 2012).

### Correlations

The current study reveals that adolescents who exhibited higher acceptance scores on the IIQ, indicating that they view autism as an integral and positive part of their identity, reported higher levels of autonomy and social participation. This positive identification with autism likely fosters greater self-confidence and a willingness to engage independently in daily and social activities, directly enhancing their overall QoL (R. Cooper et al., 2021). Conversely, adolescents with higher engulfment scores, which indicate they perceive autism as an overwhelming and dominant aspect of their identity, demonstrate lower autonomy and social participation levels, which may negatively impact their emotional well-being. This finding aligns with prior research that demonstrated adolescents who perceive their condition as engulfing may internalize societal stigma, reduced self-efficacy, heightened emotional difficulties such as anxiety or depression, and greater difficulty navigating social situations (R. S. P. Jones et al., 2014; Oris et al., 2018).

These emotional challenges, in turn, can create barriers to autonomy and participation, as adolescents may feel less capable of managing everyday tasks or engaging with peers in meaningful ways (Shochet et al., 2016). Additionally, the relationship between engulfment and lower participation suggests that adolescents who feel their autism dominates their identity may avoid social activities out of fear of rejection or misunderstanding, further limiting their opportunities for personal growth and social integration (Cage et al., 2018).

## Limitations and Recommendations for Future Research

Despite the increased awareness this study provided, several limitations should be noted. One limitation is the final sample size, which included 30 dyads. Although this sample size provides valuable insights, it falls short of the number suggested by the a priori power analysis for adequate statistical power, potentially reducing the findings’ generalizability and increasing the risk of Type II errors. Additionally, using ICC to establish agreement has limitations, particularly in small samples (Shieh, 2014). Future research should address these issues by recruiting a larger sample to ensure sufficient power and robustness of results. Combining advanced methods with more meticulous sample size planning will enhance the reliability and generalizability of findings. Furthermore, all autistic participants in the study were classified as ‘Level 1’ and did not have an intellectual disability. This limits the generalizability of the findings to autistic individuals with higher support needs or co-occurring intellectual disabilities. Future research should consider a more diverse range of autistic profiles to enhance the applicability of the results across the spectrum.

In addition, although self- and parental reports are crucial for understanding subjective and external perspectives, they may introduce biases. Objective measures like direct behavioral observations could provide a more accurate assessment. The CASP-Y questionnaire also provided valuable insights into social participation across key life domains (Bedell, 2009; McDougall et al., 2013). However, its focus on participation based on age-expected activities may not fully capture the complexity of social engagement or subjective experiences of autistic adolescents. This limitation highlights the need for measures that better reflect the nuances of social participation of autistic adolescents (Coster & Khetani, 2008). Finally, given the strong impact of engulfment on autonomy and QoL, future research should explore interventions that reduce negative autism identity feelings while promoting self-acceptance and coping strategies to enhance autistic adolescents’ functional autonomy, social participation, and QoL.

## Conclusions

This study provides valuable insights into the autism identity perceptions of autistic adolescents and the impact of these perceptions on their functional autonomy, social participation, and overall QoL. Feelings of engulfment by the autism diagnosis were linked to lower emotional and social QoL, highlighting the need for interventions that address these feelings. Fostering a positive and integrated autism identity through therapeutic approaches that promote self-acceptance and coping strategies may improve social engagement, emotional well-being, and overall life satisfaction. The moderate agreement between adolescent self-reports and parental reports highlights the potential benefit of integrating both perspectives in assessment and intervention planning, particularly in ways that directly support the adolescent’s well-being. However, it is essential to acknowledge that objective social participation and QoL measures may not fully capture the adolescent’s subjective experiences because their perceptions of well-being may differ from conventional social expectations.
